# Evaluation of formaldehyde, particulate matters 2.5 and 10 emitted to a 3D printing workspace based on ventilation

**DOI:** 10.1038/s41598-022-25957-x

**Published:** 2022-12-14

**Authors:** Taehun Kim, Dayeong Hong, Sojin Moon, Namkug Kim

**Affiliations:** 1 Department of Convergence Medicine, Asan Medical Institute of Convergence Science and Technology, Asan Medical Center, University of Ulsan College of Medicine, Seoul, Republic of Korea; 2 Department of Biomedical Engineering, Asan Medical Institute of Convergence Science and Technology, Asan Medical Center, University of Ulsan College of Medicine, Seoul, Republic of Korea; 3 Department of Radiological science, Dongnam Health University, Suwon-si, Gyeonggi-do 50 Cheoncheon-ro 74 Gil, Jangan-gu, Republic of Korea; 4 Department of Radiology, University of Ulsan College of Medicine, Asan Medical Center, Seoul, Republic of Korea

**Keywords:** Environmental impact, Preventive medicine, Biomedical engineering, Applied physics

## Abstract

Recently, the development of 3D printing (3DP) technology and its application in various fields have improved our quality of life. However, hazardous materials that affect the human body, such as formaldehyde and particulate matter (PM), are emitted into the air during 3DP. This study measured the formaldehyde, PM_10_, and PM_2.5_ emitted by 3DP with the ventilation operation using six materials in material extrusion (ME) and vat photopolymerization (VP) and compared them between the 3DP workspace and the control setting with test–retest validation by two researchers. The experiments were divided into four stages based on the 3DP and ventilation operation. A linear mixed model was used to analyze the mean differences and tendencies between the 3DP workspace and the control setting. The change as ventilation was switched from off to on was evaluated by calculating the area. The differences and tendencies were shown in the statistically significant differences from a post-hoc test (α = 0.0125) except for some cases. There was a significant difference in formaldehyde depending on the ventilation operation; however, only a minor difference in PM_10,_ and PM_2.5_ was confirmed. The amount of formaldehyde exceeding the standard was measured in all materials during 3DP without ventilation. Therefore, it is recommended to operate ventilation systems.

## Introduction

Since the development of 3D printing (3DP) technology in the 1980s, it has been used in various industries, including electronics, automobiles, aerospace, education, and medicine^1^. In the medical field, 3DP technologies have been used to create patient-specific surgical guides, simulators, surgery planning, and prosthetics to address medical doctors’ clinical unmet needs^2,3^. Rapid prototyping or additive manufacturing is a technology that includes materials extrusion (ME), vat photopolymerization (VP), and selective laser sintering; and is fabricated to the materials layer-by-layer until the object is completely built^4^. The benefit of the 3DP technology is that it is more accurate, inexpensive, and has a shorter manufacturing time than traditional subtractive manufacturing, allowing for the fabrication of complex structures^4^. Several filament materials are available, including polylactic acid (PLA), acrylonitrile butadiene styrene (ABS), thermoplastic polyurethane (TPU), polyvinyl alcohol (PVA), nylon in the ME, and resins such as Clear, Dental, and Flexible in the VP and DLP^5^. ME is a technology that builds 3D objects by heating the thermoplastic filament from the heat block in the extruder with a temperature between 180 and 270 °C and releasing the heated materials from the nozzle while pushing it with the rotational step motors. The VP is built by reflecting the ultraviolet laser generated from the source to a controlled mirror, focusing on the photosensitive resin in the tray, and curing the materials point by point on the build platform. The photosensitive resin is heated to a temperature between 20 and 70 °C, and the previous layer is wiped before the next layer is cured.

Hazardous materials, including formaldehyde and particulate matter (PM) are released during the 3DP process by melting the thermoplastic filament and heating the resin at high temperatures^6–12^. Among volatile organic compounds (VOCs), formaldehyde is formed by the oxidation of methane or methanol in the presence of a catalyst, and it is produced when a material containing carbon is incompletely burned. Furthermore, it is frequently detected in forest fires, cigarette smoke, automobile smoke, and everyday items like floor coverings and wallpaper^13^. The formaldehyde emitted during 3DP causes severe air pollution in workplaces, public places, and private homes and has a particularly negative impact on the human body^6^, and the toxic material is a flammable, colorless gas. The World Health Organization (WHO) recommends an indoor formaldehyde concentration limit^14^ of 100 µg/m^3^. PM is produced in the atmosphere by natural events such as wind, and those produced by anthropogenic activities can harm human health and environmental quality. The regulations of PM are PM ≤ 10 µm/m^3^ (PM_10_) and PM ≤ 2.5 µm/m^3^ (PM_2.5_) in diameter. The established levels of the standard for PM_10_ and PM_2.5_ are that the average concentration of exposure per year is 150 µg/m^3^ and 65 µg/m^3^ with no more than exceedance and that the concentration of average annual exposure for 3 years not to exceed^15^ 50 µg/m^3^ and 15 µg/m^3^.

Many studies have been conducted on the release of 3DP hazardous materials, which can be affected by various factors such as materials, operating temperature, 3DP technologies, and 3D models Some studies were conducted by varying the temperature depending on the material of the FDM. Jeon et al.^8^ measured the particle concentration in the four FDM materials by raising the temperature from 185 to 290 °C at 15 °C intervals. The particles emitted at the lowest and highest temperatures were 10^7^–10^9^ particles/min and 10^11^ particles/min, respectively, representing a difference of approximately 100–10,000 times. Stephens et al.^12^. The particles for the PLA and ABS were measured similarly by the ME and yielded results of ~ 2.0 × 10^10^ particles/min and ~ 1.9 × 10^11^ particles/min, respectively. Among the 3DP technologies, their study measured the number of emitted particles using only ME materials. To supplement previous research, this study measured the concentrations of formaldehyde, PM_10,_ and PM_2.5_ emitted by the ME and VP with ventilation operation and compared them between the 3DP workspace and the control setting using test–retest validation.

## Methods

### Specification of 3D printers and materials

The experiments were conducted with the help of a commercially available ME printer (Ultimaker S5, Ultimaker Inc.) with PLA, ABS, and TPU filament (Ultimaker S5, Ultimaker Inc.) and VP printer (Form3, Formlabs Inc.) with Clear, Dental LT, and Flexible 80 A resin (Form3, Formlabs Inc.). The ME extruder had a nozzle diameter of 0.4 mm, a layer resolution of 60–150 μm, and a printable area with a field of view (FOV) of 330 × 240 × 300 mm^3^. In Ultimaker Cura (Ultimaker Inc.) slicer settings, all layer thicknesses were 0.2 mm. The nozzle temperatures for PLA, ABS, and TPU filament with 2.85 ± 0.10 mm diameter were set at 200 °C, 240 °C, and 223 °C, respectively, and the plate temperatures were 60 °C for the PLA and TPU and 85 °C for the ABS as recommended by the manufacturer. The travel speed of the extruders was 150 mm/s, and the PLA print speeds were 70 mm/s, whereas the ABS and TPU print speeds were 55 and 25 mm/s, respectively. The manufacturer chose 85 μm and 250 mW for the laser spot size and power, which are components of the VP. The X–Y plane resolution was 25 μm, and the z-axis resolution was 25–300 μm, with different ranges depending on the material (Clear, Dental LT, and Flexible 80A resin). VP had a FOV of 140 × 145 × 185 mm^3^ and objects were printed with a layer thickness of 0.1 mm using the PreForm slicer (Formlabs Inc.).

### Layout of the 3DP workspace, monitoring set-up, and 3D printed test part

**Figure 1:**
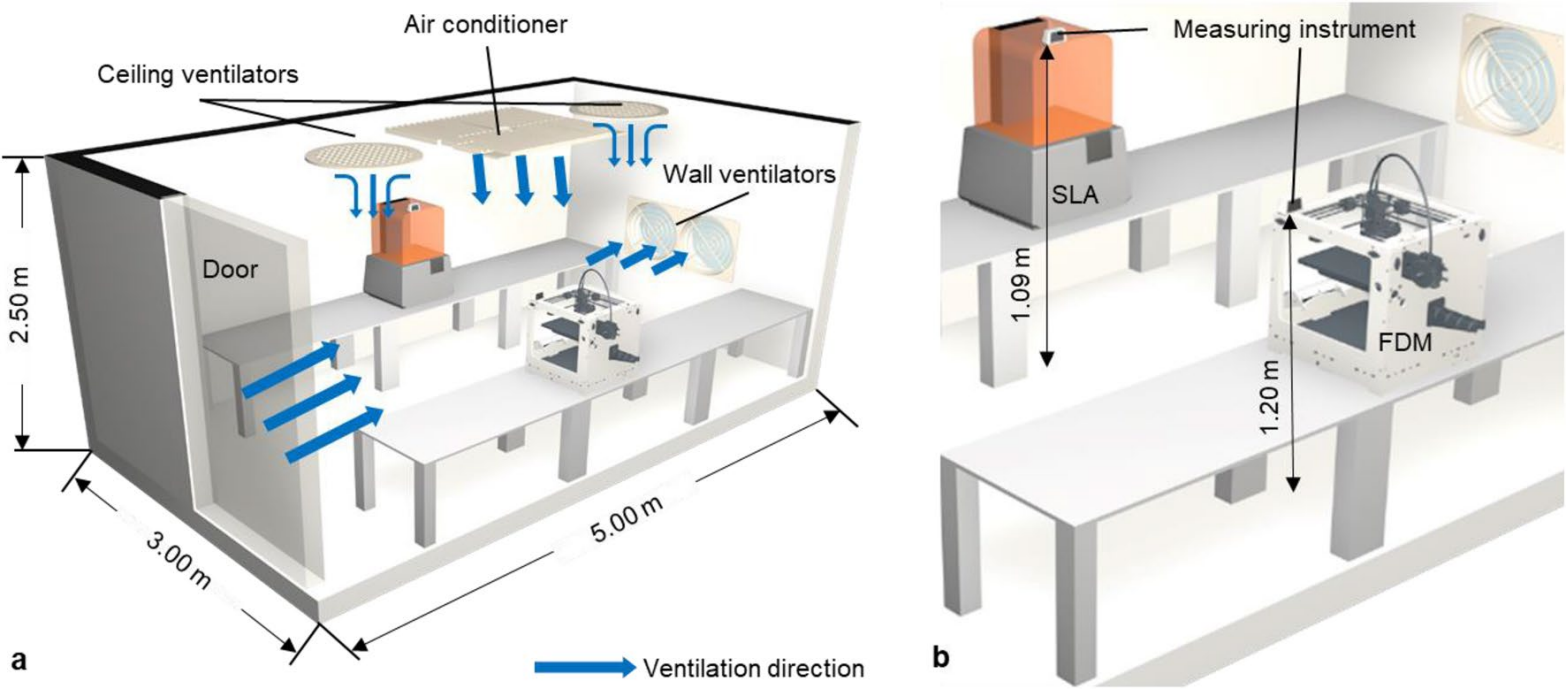
HYPERLINK "sps:id::fig1||locator::gr3||MediaObject::0" The 3D printing (3DP) workspace with ventilation facility. (**a**) The layout of the 3DP workspace. (**b**) The location of measurement.

**Figure 2:**
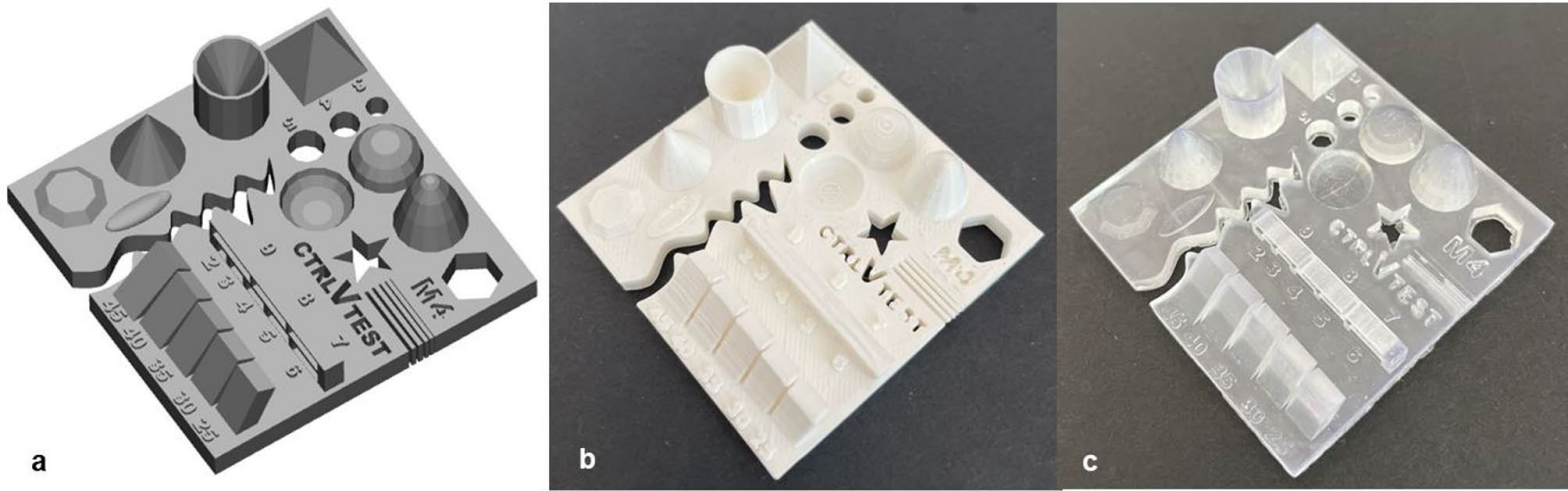
The specification of the test part. (**a**) 3D model, (**b**) a test part printed by materials extrusion (ME), and (**c**) a test part printed by vat photopolymerization (VP).

Figure 1 shows the structure and setting of the laboratory room with a ventilation facility in the 3DP workspace. The 3DP workspace was built with 3D printers, two positive-pressure ceiling ventilators and an air conditioner, two negative-pressure automatic shutters and grill-type wall ventilators, and a measuring instrument (Fig. 1a). The 3DP workspace with dimensions of 5.0 × 3.0 × 2.5 m^3^ was kept at a temperature interval of 18–22 °C. The 3DP workspace was designed in such a way that wind enter from the outside through an entrance and is cool using the air conditioner and ceiling ventilators and are emitted through the two wall ventilators. Two automatic shutter and grill-type fans mounted on the wall were rotated at 1090 revolutions per minute, with a maximum air volume of 12 m^3^/min with a power consumption of 44 W. The measuring instrument (Coamise S4, Koares Inc.) with the dimensions of 112 × 46 × 65 mm^3^ and capable of monitoring at 1-s intervals was composed of the gas sensors that measure formaldehyde and the laser detectors that can absorb scattering activities for concentrating PM and was located at the top of the 3D printers with a height of 1.20 or 1.09 m (Fig. 1b). The 3DP test part, depicted in Fig. 2, was used in the experiment for 3DP troubleshooting. This 3DP part had the following dimensions: (1) 50 × 50 × 4 mm, (2) 3 holes with 3, 4, and 5 mm, the shape of the pyramid, cone, wave, and half-sphere, (3) thin walls with intervals of 0.1 mm between 0.1 and 0.5 mm, (4) overhang with an interval of 5° between 25° and 45°, and (5) the bridge with an interval of 1.0 mm between 2.0 and 9.0 mm.

### Control setting and air quality

**Figure 3:**
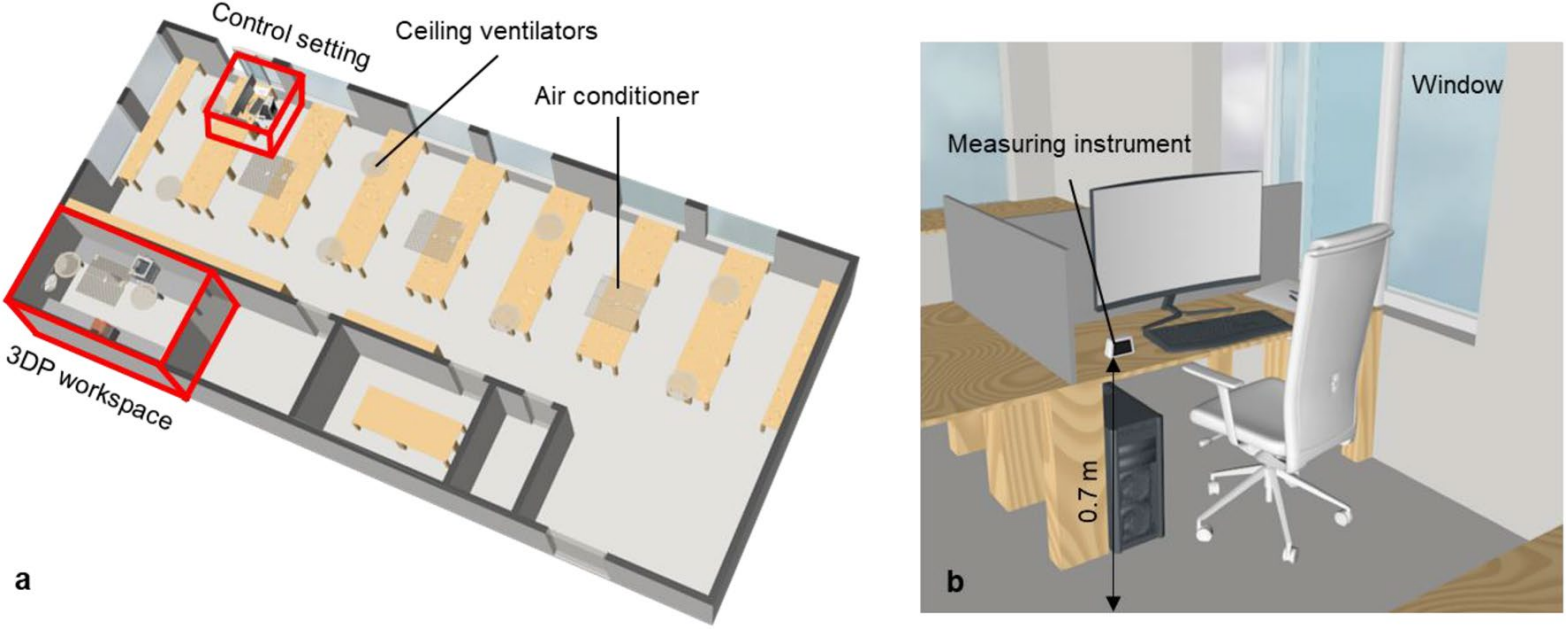
The office is the control setting. (**a**) The layout of the control setting and (**b**) the location of measurement.

The office was chosen as the control setting to confirm the hazardous materials in the 3DP workspace intuitively. The control setting was outfitted with air purifiers, wall windows, ceiling ventilators, and positive-pressure air conditioners and was meticulously managed in an environment free of stimuli such as smoking, perfume, or food (Fig. 3a). The measuring instrument was placed on a desk with a height of 0.7 m next to an open wall window in the control setting (Fig. 3b) and was simultaneously performed as the 3DP workspace experiments. After each experiment, the 3DP workspace was ventilated for approximately 30 min without any 3D printer operation to return to a consistent and clear state. Furthermore, hazardous materials in the measuring instrument’s sensor were purged for approximately 30 min before performing all experiments to avoid inaccurate measurements with sensor contamination.

### Evaluation and analysis

Measurements were taken at 1-s intervals for approximately 1 h per experiment to evaluate the formaldehyde, PM_10_, and PM_2.5_ emitted from the 3DP workspace and the control setting and were classified into four stages based on the 3DP and ventilation operation: (1) stage I is the preparation for 3DP operation without ventilation (0–2.5 min) and (2) stage II is the actual time for 3DP operation without ventilation (2.5–27.5 min), (3) stage III is the time for 3DP operation with ventilation (27.5–52.5 min), and (4) stage IV is the wash-out time for 3DP operation with ventilation (52.5–55 min). Two methods were used to evaluate the analysis: (1) the mean differences in formaldehyde, PM_10_, and PM_2.5_ levels for the ME and VP, compared to the 3DP workspace and the control setting. The statistics were applied to a linear mixed model with interaction, group, and the time effects. Multiple testing for group effect was performed using the Bonferroni post-hoc test set to α = 0.0125. (2) The change in hazardous materials emitted as ventilation is switched from off to on was calculated and digitized to Eq. (1):

1 $$Area = \sum\limits_{k = 1}^{n} {\frac{{C_{k} + C_{k - 1} }}{2}(T_{k} - T_{k - 1} )}$$

where C_k_ is the concentration of the k-th emitted formaldehyde, PM_10,_ and PM_2.5_, and T_k_ is the time. Furthermore, the test–retest procedure was used to confirm the validation of the experiments using two evaluation methods.

## Results

### Comparison between the 3DP workspace and the control setting

**Figure 4:**
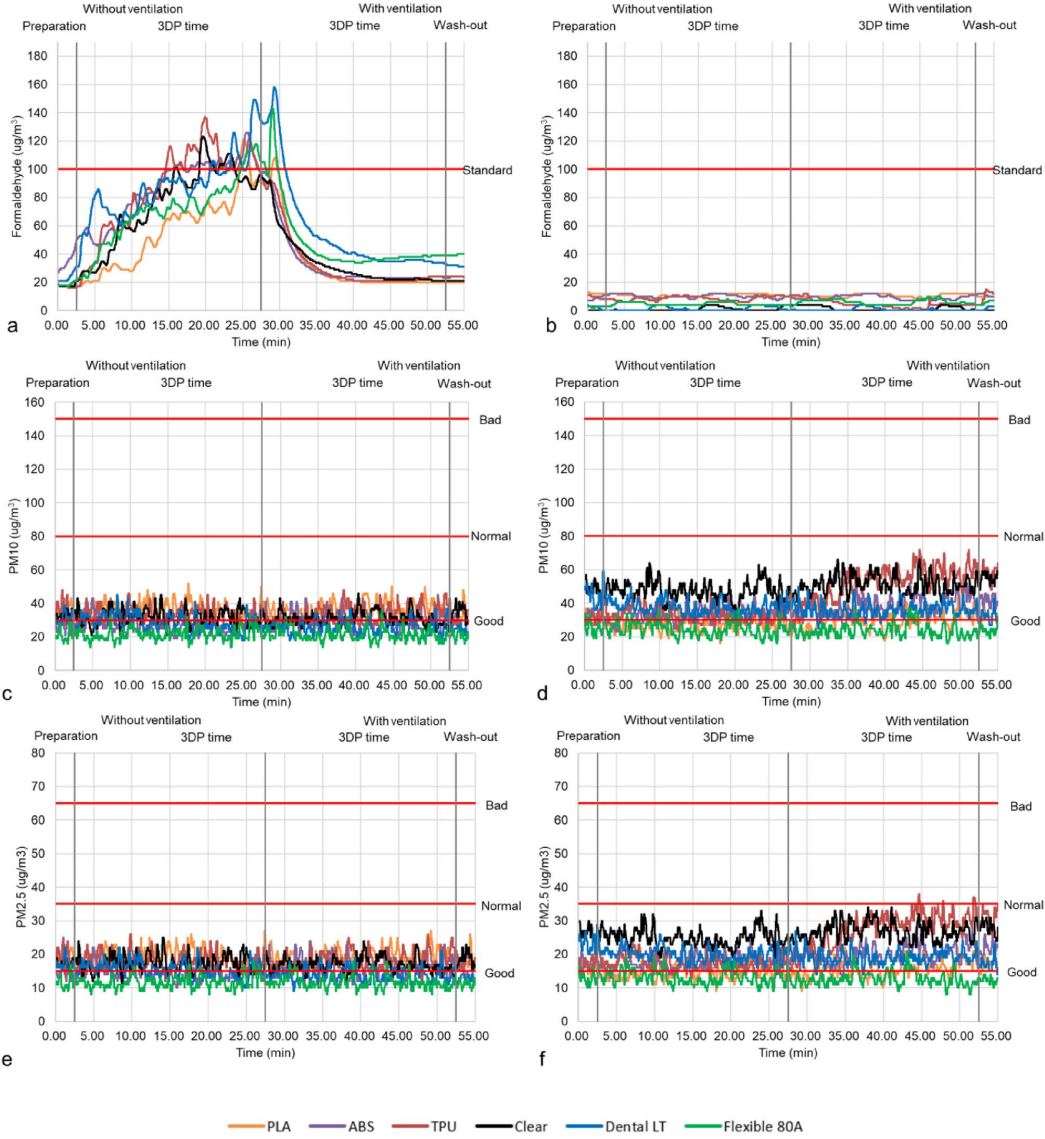
Test of emission amount for the polylactic acid (PLA), acrylonitrile butadiene styrene (ABS), and thermoplastic polyurethane (TPU) with materials extrusion (ME) and Clear, Dental LT, and Flexible 80A with the vat photopolymerization (VP) divided by 4 stages in the (**a**) 3D printing (3DP) workspace and (**b**) the control setting of formaldehyde, (**c**) the 3DP workspace and (**d**) the control setting of particulate matters 10 (PM_10_), and (**e**) the 3DP workspace and (**f**) the control setting of particulate matters 2.5 (PM_2.5_).

**Figure 5:**
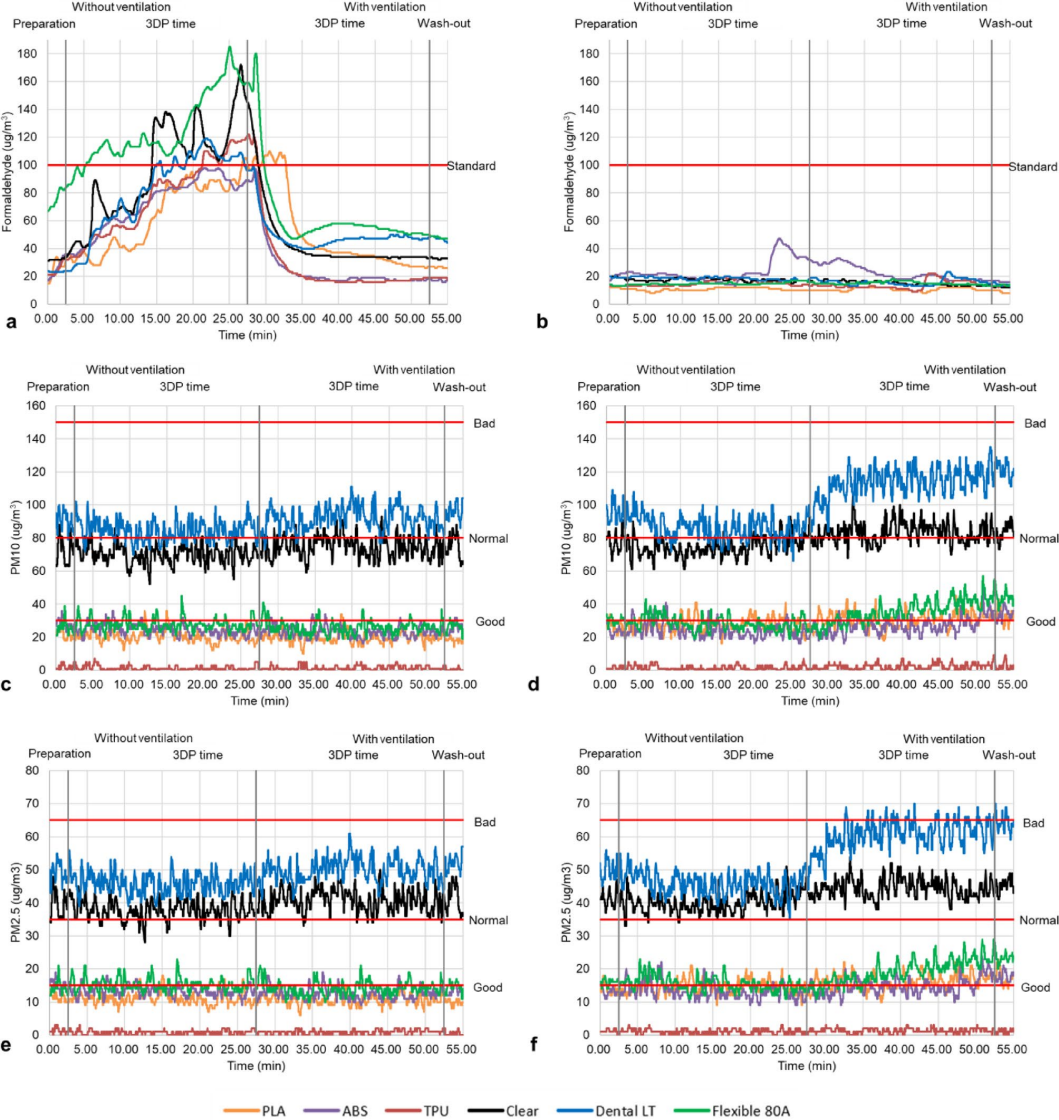
Retest of emission amount for the polylactic acid (PLA), acrylonitrile butadiene styrene (ABS), and thermoplastic polyurethane (TPU) with materials extrusion (ME) and Clear, Dental LT, and Flexible 80A with the vat photopolymerization (VP) divided by 4 stages in the (**a**) 3D printing (3DP) workspace and (**b**) the control setting of formaldehyde, (**c**) the 3DP workspace and d. the control setting of particulate matters 10 (PM_10_), and (**e**) the 3DP workspace and f. the control setting of particulate matters 2.5 (PM_2.5_).

Table 1 and Figs. 4 and 5 depict the measurement and mean differences of hazardous materials categorized into four stages in the test–retest. All the measured raw data of the test–retest is presented in Supplementary Tables S1 and S2. The comparison of the 3DP workspace and the control setting is confirmed to be a significant difference in the group effect (P < 0.001) (Supplementary Table S3); however, this was corrected using Bonferroni post-hoc after adjusting significance probability (α = 0.0125) (Table 1). The hazardous materials emitted during preparation and 3DP time without ventilation (stages I and II) were detected to be statistically significant differences except for rare cases in PLA, ABS, TPU of ME, and VP Dental LT and Flexible 80A, and 3DP time and wash-out with ventilation (stage III and IV) were confirmed to be statistically significant differences except for PM_10_ and PM_2.5_ in PLA and formaldehyde in ABS of ME through the test–retest (Table 1). Table 2 shows the ranges and grades of PM_10_ and PM_2.5_ concentrations emitted by each material. The tendency between the 3DP workspace and the control setting was more severe in formaldehyde than in PM_10_ and PM_2.5_ (Figs. 4 and 5). Nevertheless, statistically significant differences were demonstrated in all experiments (P < 0.001 or *P* < 0.01) (Supplementary Table S3).

**Table 1:** The differences in formaldehyde, PM_10_, and PM_2.5_ emissions between the 3DP workspaces and control settings for the PLA, ABS, and TPU of the ME and the Clear, Dental LT, and Flexible 80A resin of the VP in four stages with test–retest. *< 0.001, **< 0.05, ***< 0.01.

Measurement (µg/m^3^)				Without ventilation						With ventilation					
				Preparation			3DP time			3DP time			Wash-out		
				3DP workspace	Control setting	Difference (p)	3DP workspace	Control setting	Difference (p)	3DP workspace	Control setting	Difference (p)	3DP workspace	Control setting	Difference (p)
Test	ME	PLA	Formaldehyde	17.65 ± 0.47	12.10 ± 0.30	5.54 ± 0.65**	56.10 ± 26.20	10.85 ± 1.05	45.2 ± 26.0*	33.66 ± 24.40	10.66 ± 1.15	22.9 ± 24.8*	20.00 ± 0.00	10.10 ± 0.30	9.9 ± 0.30*
			PM_10_	36.38 ± 2.88	31.92 ± 3.81	5.01 ± 3.77*	35.83 ± 4.55	27.50 ± 4.10	8.8 ± 5.94*	36.31 ± 4.69	30.36 ± 5.05	7.38 ± 5.26*	32.20 ± 4.77	32.91 ± 2.86	3.58 ± 3.46 (1.000)
			PM_2.5_	19.26 ± 1.65	16.35 ± 1.83	2.97 ± 2.06*	86.16 ± 22.00	10.22 ± 1.27	4.64 ± 3.00*	33.56 ± 20.40	9.20 ± 1.63	4.11 ± 2.68*	23.77 ± 0.42	11.12 ± 0.86	2.09 ± 1.78 (0.195)
		ABS	Formaldehyde	34.86 ± 5.62	9.05 ± 1.54	25.8 ± 4.28*	86.16 ± 22.00	10.22 ± 1.27	75.9 ± 22.4*	33.56 ± 20.40	9.20 ± 1.63	24.3 ± 20.6*	23.77 ± 0.42	11.12 ± 0.86	12.6 ± 1.08*
			PM_10_	26.68 ± 4.32	32.76 ± 3.41	7.18 ± 4.20*	26.81 ± 4.22	33.56 ± 4.32	7.89 ± 5.11*	29.31 ± 5.21	38.61 ± 5.56	10.2 ± 6.26*	28.18 ± 4.30	42.37 ± 3.02	14.1 ± 5.50*
			PM_2.5_	14.80 ± 2.36	17.39 ± 1.45	3.50 ± 1.98*	14.62 ± 2.14	17.64 ± 2.06	3.61 ± 2.51*	15.98 ± 2.79	20.03 ± 2.75	4.79 ± 3.27*	15.22 ± 2.34	22.06 ± 1.91	6.84 ± 3.23*
		TPU	Formaldehyde	16.80 ± 0.81	9.28 ± 1.13	7.51 ± 0.50*	86.40 ± 30.40	8.59 ± 1.69	77.8 ± 30.2*	33.90 ± 21.30	4.73 ± 2.74	29.1 ± 18.8*	23.99 ± 0.08	9.14 ± 4.34	14.8 ± 4.34*
			PM_10_	35.25 ± 5.70	32.47 ± 4.38	6.42 ± 4.67*	33.22 ± 4.41	33.55 ± 4.81	4.67 ± 4.01 (0.657)	33.62 ± 5.12	52.16 ± 9.62	18.8 ± 8.88*	32.72 ± 4.28	57.61 ± 4.00	24.8 ± 7.08*
			PM_2.5_	19.00 ± 2.93	17.41 ± 2.24	3.31 ± 2.59*	17.99 ± 2.46	17.59 ± 2.28	2.37 ± 2.03***	18.24 ± 2.62	27.55 ± 4.96	9.49 ± 4.69*	17.14 ± 1.76	30.48 ± 2.75	13.3 ± 3.79*
	VP	Clear	Formaldehyde	17.35 ± 0.94	0.00 ± 0.00	15.9 ± 4.79*	75.32 ± 27.80	0.85 ± 1.42	74.4 ± 27.3*	34.45 ± 18.80	1.334 ± 1.71	33.1 ± 17.8*	21.00 ± 0.00	0.00 ± 0.00	21.00 ± 0.00*
			PM_10_	33.06 ± 2.53	48.40 ± 3.04	14.1 ± 5.36*	31.95 ± 4.80	47.53 ± 5.31	15.6 ± 7.33*	30.85 ± 4.03	51.15 ± 5.50	20.3 ± 7.72*	34.79 ± 4.35	54.97 ± 2.88	20.10 ± 5.20*
			PM_2.5_	17.35 ± 0.97	26.33 ± 1.67	8.26 ± 3.00*	17.34 ± 2.53	24.99 ± 2.57	7.67 ± 3.76*	16.78 ± 2.10	26.77 ± 2.75	9.99 ± 3.92*	18.73 ± 1.98	27.97 ± 2.01	9.24 ± 2.27*
		Dental LT	Formaldehyde	23.49 ± 3.32	1.99 ± 1.31	21.5 ± 4.57*	87.33 ± 21.90	1.18 ± 1.53	86.1 ± 21.8*	56.39 ± 33.40	0.56 ± 1.16	55.8 ± 33.6*	31.68 ± 0.51	1.31 ± 1.37	30.30 ± 1.84*
			PM_10_	31.29 ± 3.51	44.13 ± 5.70	13.0 ± 7.93*	28.42 ± 4.73	37.58 ± 4.29	9.43 ± 5.75*	25.60 ± 4.07	36.00 ± 3.98	10.5 ± 4.95*	24.34 ± 2.09	32.50 ± 3.93	8.22 ± 4.86*
			PM_2.5_	16.78 ± 1.96	23.31 ± 2.28	6.65 ± 3.16*	15.37 ± 2.37	19.91 ± 2.08	4.70 ± 2.67*	13.80 ± 2.01	19.15 ± 1.97	5.39 ± 2.40*	12.76 ± 0.96	17.28 ± 1.92	4.52 ± 2.12*
		Flexible 80A	Formaldehyde	18.71 ± 1.28	2.81 ± 0.39	15.9 ± 1.24*	70.15 ± 23.00	4.66 ± 1.08	65.4 ± 23.3*	48.45 ± 24.10	5.97 ± 1.75	42.4 ± 24.0*	39.38 ± 0.48	5.35 ± 1.30	34.0 ± 0.91*
			PM_10_	21.52 ± 4.02	24.86 ± 3.68	4.21 ± 2.96*	21.75 ± 3.40	24.57 ± 4.15	4.63 ± 3.46*	21.49 ± 3.74	23.73 ± 4.09	4.54 ± 3.44*	20.78 ± 2.37	23.58 ± 2.41	3.59 ± 2.48*
			PM_2.5_	11.74 ± 2.17	12.81 ± 1.55	1.52 ± 1.27*	11.70 ± 1.77	13.13 ± 2.09	2.28 ± 1.76*	11.61 ± 1.82	12.66 ± 2.05	2.09 ± 1.83*	11.14 ± 1.14	12.20 ± 0.90	1.34 ± 1.15*
Retest	ME	PLA	Formaldehyde	26.04 ± 6.29	11.60 ± 0.48	13.9 ± 6.48*	62.93 ± 24.40	10.69 ± 1.04	52.0 ± 23.9*	51.55 ± 29.00	10.67 ± 1.15	40.8 ± 29.4*	26.58 ± 0.49	9.05 ± 0.94	16.5 ± 0.53*
			PM_10_	21.67 ± 3.12	26.31 ± 3.17	5.02 ± 2.73*	20.65 ± 3.73	27.42 ± 4.17	7.28 ± 4.39*	19.83 ± 3.35	31.10 ± 4.77	11.3 ± 5.70*	18.17 ± 1.33	32.80 ± 4.52	14.6 ± 4.50*
			PM_2.5_	11.35 ± 1.01	14.19 ± 1.43	2.84 ± 1.49*	11.15 ± 1.86	14.51 ± 2.01	3.62 ± 2.15*	10.72 ± 1.62	16.23 ± 2.35	5.54 ± 2.71*	9.97 ± 0.84	17.26 ± 2.36	7.28 ± 2.27 (0.195)
		ABS	Formaldehyde	25.56 ± 6.42	20.77 ± 1.64	4.98 ± 4.89**	71.87 ± 19.20	23.61 ± 8.41	48.2 ± 17.6*	26.29 ± 19.0	22.35 ± 5.20	7.07 ± 15.2*	17.05 ± 0.66	16.42 ± 0.49	0.63 ± 0.71 (1.000)
			PM_10_	28.20 ± 2.92	23.53 ± 2.75	5.03 ± 4.04*	24.84 ± 3.30	24.10 ± 4.47	3.95 ± 3.14*	23.28 ± 3.45	26.88 ± 5.10	5.57 ± 4.66*	25.11 ± 3.45	34.08 ± 3.42	8.97 ± 3.69*
			PM_2.5_	15.07 ± 1.61	13.23 ± 1.30	2.07 ± 2.04*	13.40 ± 1.54	13.26 ± 2.35	1.92 ± 1.63 (0.24)	12.58 ± 1.74	14.58 ± 2.54	2.87 ± 2.39*	13.12 ± 1.11	17.60 ± 1.54	4.47 ± 1.38*
		TPU	Formaldehyde	24.82 ± 4.51	13.00 ± 0.00	11.8 ± 4.51*	76.33 ± 27.10	15.01 ± 1.99	61.3 ± 26.3*	27.96 ± 25.50	13.82 ± 3.01	15.0 ± 25.4*	18.96 ± 0.19	12.72 ± 0.45	6.24 ± 0.44*
			PM_10_	2.06 ± 1.53	1.64 ± 0.93	0.82 ± 1.05 (0.050)	1.29 ± 1.34	1.493 ± 1.41	1.52 ± 1.42*	1.43 ± 1.29	2.35 ± 1.74	1.79 ± 1.56*	0.56 ± 0.96	2.28 ± 2.45	2.21 ± 2.39*
			PM_2.5_	1.38 ± 0.78	1.23 ± 0.46	0.43 ± 0.54 (0.206)	0.78 ± 0.64	1.013 ± 0.73	0.81 ± 0.71*	0.88 ± 0.63	1.29 ± 0.70	0.75 ± 0.67*	0.45 ± 0.69	1.05 ± 0.70	1.05 ± 0.73*
	VP	Clear	Formaldehyde	32.21 ± 0.68	19.21 ± 0.90	12.9 ± 1.51*	96.75 ± 35.40	17.49 ± 0.99	79.2 ± 35.6*	44.42 ± 23.50	15.07 ± 1.52	29.3 ± 23.0*	32.96 ± 0.54	12.60 ± 0.78	20.3 ± 0.87*
			PM_10_	72.09 ± 7.76	77.45 ± 5.77	9.53 ± 5.77*	70.24 ± 5.63	74.83 ± 5.73	7.33 ± 5.55*	74.98 ± 6.47	83.25 ± 6.12	10.0 ± 6.58*	75.33 ± 7.16	84.22 ± 5.04	9.97 ± 8.12*
			PM_2.5_	39.56 ± 3.93	41.27 ± 2.41	4.57 ± 2.26*	37.86 ± 3.04	40.02 ± 2.96	3.67 ± 2.87*	40.77 ± 3.28	44.44 ± 2.91	4.73 ± 3.14*	41.38 ± 4.07	45.58 ± 2.07	4.84 ± 3.80*
		Dental LT	Formaldehyde	23.20 ± 0.40	19.19 ± 0.40	4.00 ± 0.48 (0.074)	81.32 ± 28.40	18.50 ± 1.04	62.8 ± 29.0*	49.53 ± 12.00	15.93 ± 2.39	33.6 ± 11.4*	47.14 ± 1.48	14.07 ± 1.01	33.0 ± 1.02*
			PM_10_	90.50 ± 5.57	95.33 ± 6.19	7.59 ± 6.06*	84.79 ± 6.18	86.58 ± 7.06	7.47 ± 5.67*	92.02 ± 7.05	113.80 ± 8.94	22.1 ± 10.7*	96.10 ± 4.85	121.10 ± 4.80	25.0 ± 5.74*
			PM_2.5_	49.16 ± 2.96	49.87 ± 2.93	3.11 ± 2.52 (0.354)	45.92 ± 3.14	45.91 ± 3.39	3.6 ± 2.67 (1.000)	49.87 ± 3.55	60.66 ± 4.49	10.9 ± 5.31*	52.10 ± 2.41	64.16 ± 2.46	12.0 ± 2.68*
		Flexible 80A	Formaldehyde	75.84 ± 6.29	13.26 ± 0.44	62.5 ± 6.37*	125.30 ± 24.90	14.85 ± 0.91	110. ± 24.3*	64.22 ± 28.70	15.16 ± 1.13	49.0 ± 28.7*	47.90 ± 0.86	13.36 ± 0.48	34.5 ± 1.20*
			PM_10_	25.96 ± 4.04	30.28 ± 2.13	5.70 ± 3.32*	27.69 ± 3.70	28.34 ± 4.18	4.15 ± 3.65*	25.83 ± 3.90	35.59 ± 7.00	10.8 ± 6.55*	26.26 ± 2.94	43.72 ± 3.62	17.4 ± 4.82*
			PM_2.5_	14.28 ± 2.03	16.03 ± 0.93	2.57 ± 1.51*	15.26 ± 1.97	15.46 ± 2.16	2.24 ± 1.93 (0.119)	14.17 ± 2.06	19.17 ± 3.58	5.51 ± 3.39*	14.50 ± 1.44	23.29 ± 1.44	8.79 ± 2.27*

*3DP* 3D printing, *ME* materials extrusion, *PLA* polylactic acid, *PM*_*10*_ particulate matter 10, PM_2.5_ particulate matter 2.5, *ABS* acrylonitrile butadiene styrene, *TPU* thermoplastic polyurethane, *VP* vat photopolymerization.

**Table 2:** The ranges and grades for the concentrations of PM_10_ and PM_2.5_ with ME and VP with test and retest.

			PM_10_				PM_2.5_			
			Control setting		3DP workspace		Control setting		3DP workspace	
			Range (µg/m^3^)	Grade*	Range (µg/m^3^)	Grade*	Range (µg/m^3^)	Grade**	Range (µg/m^3^)	Grade**
Test	ME	PLA	16.00–45.00	Good–Normal	23.00–52.00	Good–Normal	9.00–22.00	Good–Normal	11.00–27.00	Good–Normal
		ABS	23.00–54.00		18.00–45.00		13.00–28.00		10.00–24.00	
		TPU	23.00–72.00		21.00–48.00		13.00–38.00	Good–Bad	12.00–26.00	
	VP	Clear	34.00–66.00	Good–Normal	19.00–46.00	Good–Normal	19.00–34.00	Normal	11.00–25.00	Good–Normal
		Dental LT	25.00–59.00		18.00–48.00		14.00–29.00	Good–Normal	9.00–22.00	
		Flexible 80A	16.00–37.00		14.00–36.00		8.00–21.00		8.00–18.00	
Retest	ME	PLA	16.00–45.00	Good–Normal	10.00–36.00	Good–Normal	9.00–23.00	Good–Normal	6.00–18.00	Good–Normal
		ABS	16.00–50.00		16.00–36.00		9.00–24.00		9.00–19.00	
		TPU	0.00–9.00	Good	0.00–7.00	Good	0.00–3.00	Good	0.00–3.00	Good
	VP	Clear	61.00–106.00	Normal–Bad	52.00–93.00	Normal–Bad	33.00–54.00	Normal–Bad	28.00–50.00	Normal–Bad
		Dental	66.00–135.00		70.00–111.00		35.00–70.00		39.00–61.00	Bad
		Flexible 80A	19.00–57.00	Good–Normal	18.00–45.00	Good–Normal	11.00–29.00	Normal	10.00–23.00	Good–Normal

*PM*_*10*_ particulate matter 10, *PM*_*2.5*_ particulate matter 2.5, *3DP* 3D printing, *ME* materials extrusion, *PLA* polylactic acid, *ABS *acrylonitrile butadiene styrene, *TPU* thermoplastic polyurethane, *VP* vat photopolymerization.

*The PM_10_ standard is classified as follows: (1) Good: 0.00–30.00 μg/m^3^, (2) Normal: 31.00–80.00 μg/m^3^, and (3) Bad: 81.00–150.00 μg/m^3^.

**The PM_2.5_ standard is classified as follows: (1) Good: 0.00–15.00 μg/m^3^, (2) Normal: 16.00–35.00 μg/m^3^, and (3) Bad: 36.00–65.00 μg/m^3^.

### Comparison of hazardous materials emission based on ventilation in the 3DP workspace

The hazardous materials that change depending on whether the ventilation is working (stages I and II) or not (stages III and IV) were compared in the 3DP workspace (Table 3). The formaldehyde area differences ranged from 283.22 to 1343.04 µg × min/m^3^ in the test and 490.43–1598.27 µg × min/m^3^ in the retest, whereas the PM_10_ and PM_2.5_ had relatively small differences ranging from − 66.16 to 46.87 µg × min/m^3^ in the test and − 195.09 to 87.84 µg × min/m^3^ in the retest. Figures 4 and 5a,c,e depict the change in hazardous materials emitted as ventilation switches off to on a test–retest.

**Table 3:** The comparison of the area for emission amount based on ventilation in the 3D workspace.

3DP workspace (µg × min/m^3^)			Test			Retest		
			Ventilation off	Ventilation on	Difference	Ventilation off	Ventilation on	Difference
ME	PLA	Formaldehyde	1447.73	892.52	555.21	1639.63	1356.41	283.22
		PM_10_	987.43	988.84	− 1.41	570.87	541.67	29.19
		PM_2.5_	527.31	527.88	− 0.58	307.42	293.23	14.18
	ABS	Formaldehyde	2242.52	899.44	1343.07	1861.77	700.82	1160.95
		PM_10_	737.59	803.75	− 66.16	692.07	645.20	46.87
		PM_2.5_	403.00	437.98	− 34.98	373.02	347.69	25.32
	TPU	Formaldehyde	2202.99	908.63	1294.36	1971.78	747.59	1224.19
		PM_10_	919.39	922.92	− 3.53	37.41	37.19	0.22
		PM_2.5_	497.76	499.21	− 1.45	22.94	23.06	− 0.12
VP	Clear	Formaldehyde	1924.03	914.72	1009.31	2500.82	1194.63	1306.18
		PM_10_	875.67	858.99	16.68	1937.71	2064.13	− 126.42
		PM_2.5_	473.91	466.76	7.15	1046.11	1123.39	− 77.28
	Dental LT	Formaldehyde	2243.45	1490.37	753.08	2092.22	1357.40	734.82
		PM_10_	789.29	701.45	87.84	2347.43	2542.53	− 195.09
		PM_2.5_	426.47	377.20	49.27	1271.78	1378.08	− 106.30
	Flexible 80A	Formaldehyde	1801.57	1311.13	490.43	3325.32	1727.05	1598.27
		PM_10_	598.15	589.68	8.47	757.65	711.87	45.78
		PM_2.5_	322.08	318.28	3.80	417.62	390.75	26.87

*3DP *3D printing, *ME* materials extrusion, *PLA* polylactic acid, *PM*_*10*_ particulate matter 10, *PM*_*2.5*_ particulate matter 2.5, *ABS* acrylonitrile butadiene styrene, *TPU* thermoplastic polyurethane, *VP* vat photopolymerization.

## Discussion

With the recent development of 3DP technology, the debate over the dangers of 3DP technology has raged on. Several studies have been published in this regard, but the majority have focused on the number of emitted particles and VOCs when using materials for ME among 3DP technologies^8–10,12^. To supplement previous research, this study measured the amount of formaldehyde released and the concentrations of PM_10_ and PM_2.5_ for ME and VP based on ventilation and compared them between the 3DP workspace and the control group. As the number of individual 3DP operations and student education grows, it is critical to understand the harmful effects of 3DP and plan various preventive measures. This study evaluated the mean differences for hazardous materials, such as formaldehyde, PM_10_, and PM_2.5_ emitted between the 3DP workspace and the control setting. The change in hazardous materials emitted as ventilation was switched off to on was calculated. Furthermore, the test–retest procedure was used to confirm the validation and robustness of the experiments using two evaluation methods.

The measurements of formaldehyde, PM_10_, and PM_2.5_ were conducted using test–retest with the PLA, ABS, and TPU of the ME and the Clear, Dental LT, and Flexible 80A of the VP. In tests, the formaldehyde concentration in the 3DP workspace using most ME and VP materials exceeded the WHO recommendation of 100.00 µg/m^3^ for indoor formaldehyde^14^. However, the control setting was lower than the guideline. The results of the formaldehyde retest were similar to those of the original test. Each experiment was approximately twofold to thirty-ninefold higher than the control setting measured simultaneously. Unfortunately, all measurements exceeded the WHO recommendation of 100.00 µg/m^3^, except for ABS of ME in the retest, which was very close to the standard. In both tests and retests, the PM_10_ and PM_2.5_ emitted from the materials were measured relatively similarly or at a higher control setting compared to formaldehyde (Table 1, Figs. 4c–f, 5c–f). This could be because the 3DP workspace, an enclosed space, is less affected than the control setting that is exposed to the outside world. Therefore, this phenomenon implies that the impact of South Korea's external environment is more significant than PM_10_ and PM_2.5_ generated in the 3DP workspace. The formaldehyde concentration varies significantly depending on whether the ventilation is operating (stages I and II) or not (stages III and IV), and it descends dramatically after the transition time (Table 1, Figs. 4a, 5a). The transition time denotes the point at which hazardous materials still present in the 3DP workspace begins to be removed after ventilation is activated. In tests, the area differences for formaldehyde in the 3DP workspace ranged from 490.43 to 1343.07 µg × min/m^3^ and they were approximately 1.37–2.49 times higher than those in the control group measured simultaneously. The transition times for most of the ME and VP materials were comparable. The retest ranged from 283.22–1598.27 µg × min/m^3^ and approximately 1.21–2.66 times (Table 3). In retest, transition times were also similar for each material. The PM_10_ and PM_2.5_ emitted from the materials were similar regardless of ventilation in all materials with test and retest (Table 1, Figs. 4c,e, 5c,e).

The purpose of the control setting is to covary out the baseline level of hazardous materials in room air in South Korea. Therefore, a controlled setting needs to be measured and compared to accurately evaluate the hazardous materials generated in the 3DP workspace. The control setting was measured without artificial adjustments to reflect the real lifetime and space. The control setting is arranged in an office space with large windows on two sides, and the windows were occasionally open and closed during the experiments. It has about five times the area of the 3DP workspace, and many people are working there. Personal items such as smoking, perfume, and food were strictly controlled. Still, the movement of the people involved and the installation and operation of various electronic devices, such as computers, could not be strictly controlled.

This study had some limitations. First, this study was evaluated only using three metrics: formaldehyde, PM_10_, and PM_2.5_. Other toxic substances may be produced depending on the 3DP technologies or materials used. Additionally, this experiment was attempted only under specific conditions for the 3DP workspace, including the number of fans and types of 3D printers. Another limitation is that 3D printing settings with various parameters including shapes, temperature, and materials are limited. In future work, we need to extend this study with more configurations. Second, depending on the layout of the 3DP workspace, the structure of the ventilation system, and the chamber type, air pollution may appear differently. Furthermore, the VP depends on the installed resin's location and its filter, and the surrounding air's contamination manifests differently. Finally, further study is required to determine the location and number of optimized AC, fans, and 3D printers by confirming the hazardous materials emitted from the 3D printers with the CFD airflow simulation.

## Conclusion

During 3DP without ventilation, formaldehyde was detected in all materials and it exceeded the international standard. However, it was confirmed that the formaldehyde concentration significantly decreased with ventilation performed during test–retest validation. Therefore, installing and operating the ventilation systems in a facility equipped with 3D printers is recommended.

## Supplementary Information

Supplementary Table S1.

Supplementary Table S2.

Supplementary Table S3.

## Data Availability

All data generated or analyzed during this study are included in this published article and its Supplementary Information files.
