# Supplementary material for: Evaluation of formaldehyde, particulate matters 2.5 and 10 emitted to a 3D printing workspace based on ventilation
Source: Sci Rep. 2022 Dec 14;12:21638. doi: 10.1038/s41598-022-25957-x (PMC9750962; doi:10.1038/s41598-022-25957-x)
Supplement: Supplementary file 3 — Supplementary Table S3. [file 41598_2022_25957_MOESM3_ESM.docx]

**Supplementary Table S3.** The comparison between 3DP workspace and control setting using linear mixed model with test-restest. Note: fuse deposition modeling, FDM; stereolithography apparatus, SLA; Polylactic acid, PLA; Acrylonitrile butadiene styrene, ABS; Thermoplastic polyurethane, TPU; 3D printing, 3DP; PM; particle matter.

| Linear mixed model | | | | Group effect | Time effect | Interaction |
| --- | --- | --- | --- | --- | --- | --- |
| Test | FDM | PLA | Formaldehyde | <0.001 | <0.001 | <0.001 |
|  |  |  | PM10 | <0.001 | <0.001 | <0.001 |
|  |  |  | PM2.5 | <0.001 | <0.001 | <0.001 |
|  |  | ABS | Formaldehyde | <0.001 | <0.001 | <0.001 |
|  |  |  | PM10 | <0.001 | <0.001 | <0.001 |
|  |  |  | PM2.5 | <0.001 | <0.001 | <0.001 |
|  |  | TPU | Formaldehyde | <0.001 | <0.001 | <0.001 |
|  |  |  | PM10 | <0.001 | <0.001 | <0.001 |
|  |  |  | PM2.5 | <0.001 | <0.001 | <0.001 |
|  | SLA | Clear | Formaldehyde | <0.001 | <0.001 | <0.001 |
|  |  |  | PM10 | <0.001 | <0.001 | <0.001 |
|  |  |  | PM2.5 | <0.001 | <0.001 | <0.001 |
|  |  | Dental LT | Formaldehyde | <0.001 | <0.001 | <0.001 |
|  |  |  | PM10 | <0.001 | <0.001 | <0.001 |
|  |  |  | PM2.5 | <0.001 | <0.001 | <0.001 |
|  |  | Flexible 80A | Formaldehyde | <0.001 | <0.001 | <0.001 |
|  |  |  | PM10 | <0.001 | <0.001 | <0.01 |
|  |  |  | PM2.5 | <0.001 | <0.001 | <0.01 |
| Retest | FDM | PLA | Formaldehyde | <0.001 | <0.001 | <0.001 |
|  |  |  | PM10 | <0.001 | <0.001 | <0.001 |
|  |  |  | PM2.5 | <0.001 | <0.001 | <0.001 |
|  |  | ABS | Formaldehyde | <0.001 | <0.001 | <0.001 |
|  |  |  | PM10 | <0.001 | <0.001 | <0.001 |
|  |  |  | PM2.5 | <0.001 | <0.001 | <0.001 |
|  |  | TPU | Formaldehyde | <0.001 | <0.001 | <0.001 |
|  |  |  | PM10 | <0.001 | <0.001 | <0.001 |
|  |  |  | PM2.5 | <0.001 | <0.001 | <0.001 |
|  | SLA | Clear | Formaldehyde | <0.001 | <0.001 | <0.001 |
|  |  |  | PM10 | <0.001 | <0.001 | <0.001 |
|  |  |  | PM2.5 | <0.001 | <0.001 | <0.001 |
|  |  | Dental LT | Formaldehyde | <0.001 | <0.001 | <0.001 |
|  |  |  | PM10 | <0.001 | <0.001 | <0.001 |
|  |  |  | PM2.5 | <0.001 | <0.001 | <0.001 |
|  |  | Flexible 80A | Formaldehyde | <0.001 | <0.001 | <0.001 |
|  |  |  | PM10 | <0.001 | <0.001 | <0.001 |
|  |  |  | PM2.5 | <0.001 | <0.001 | <0.001 |
